# CompariPSSM: a PSSM–PSSM comparison tool for motif-binding determinant analysis

**DOI:** 10.1093/bioinformatics/btae644

**Published:** 2024-10-29

**Authors:** Ifigenia Tsitsa, Izabella Krystkowiak, Norman E Davey

**Affiliations:** Division of Cancer Biology, The Institute of Cancer Research, 237 Fulham Road, London, SW3 6JB, United Kingdom; Division of Cancer Biology, The Institute of Cancer Research, 237 Fulham Road, London, SW3 6JB, United Kingdom; Division of Cancer Biology, The Institute of Cancer Research, 237 Fulham Road, London, SW3 6JB, United Kingdom

## Abstract

**Motivation:**

Short linear motifs (SLiMs) are compact functional modules that mediate low-affinity protein–protein interactions. SLiMs direct the function of many dynamic signalling and regulatory complexes playing a central role in most biological processes of the cell. Motif-binding determinants describe the contribution of each residue in a motif-containing peptide to the affinity and specificity of binding to the motif-binding partner. Motif-binding determinants are generally defined as a motif consensus pattern or a position-specific scoring matrix (PSSM) encoding quantitative preferences. Motif-binding determinant comparison is an important motif analysis task and can be applied to motif annotation, classification, clustering, discovery and benchmarking. Currently, binding determinant comparison is generally performed by analysing consensus similarity; however, this ignores important quantitative information in both the consensus and non-consensus positions.

**Results:**

We have created a new tool, CompariPSSM, that quantifies the similarity between motif-binding determinants using sliding window PSSM–PSSM comparison and scores PSSM similarity using a randomisation-based probabilistic framework. The tool has been benchmarked on curated data from the eukaryotic linear motif database and experimental data from proteomic peptidephage display. CompariPSSM can be used for peptide classification to validate motif classes, peptide clustering to group functionally related conserved disordered regions, and benchmarking experimental motif discovery methods.

**Availability and implementation:**

CompariPSSM is available at https://slim.icr.ac.uk/projects/comparipssm.

## 1 Introduction

Short linear motifs (SLiMs) are short peptide regions that mediate protein–protein interactions (Davey *et al.* 2012, Tompa *et al.* 2014, Van Roey *et al.* 2014, Ivarsson and Jemth 2019). SLiMs are generally encoded by protein regions of 3–12 consecutive amino acids; however, usually only 2–4 positions account for the majority of the affinity and specificity of the interaction (Davey *et al.* 2012, Tompa *et al.* 2014). These key residues within the motif are called binding determinants and are generally conserved in all instances of the motif as they are highly complementary to the organisation and physicochemical properties of the SLiM-binding pocket (Davey *et al.* 2012, Chagoyen *et al.* 2016, Davey and Morgan 2016, Kumar *et al.* 2022). The eukaryotic linear motif (ELM) database, a repository of experimentally validated SLiM instances categorised into motif classes by motif-binding pocket (Kumar *et al.* 2022), has gathered a wealth of information on motif-binding determinants. The ELM resource has defined the binding determinants by analysing collections of experimentally validated motifs binding the same motif-binding pocket or from an evolutionary analysis of these instances. Using this approach, they defined binding determinants for each motif class describing them using a motif consensus. Furthermore, numerous high-throughput experimental approaches are now being applied to motif discovery and characterisation, and these analyses are producing extensive information on motif instances and motif-binding pocket-binding determinants (Younger *et al.* 2017, Nguyen *et al.* 2019, Bandyopadhyay *et al.* 2020, Hwang *et al.* 2021, Sanborn *et al.* 2021, Davey *et al.* 2022, 2023, Shi *et al.* 2023). Consequently, high-quality binding determinant information from peptide arrays, phage display and deep mutational scanning is becoming increasingly common.

**Figure 1.**
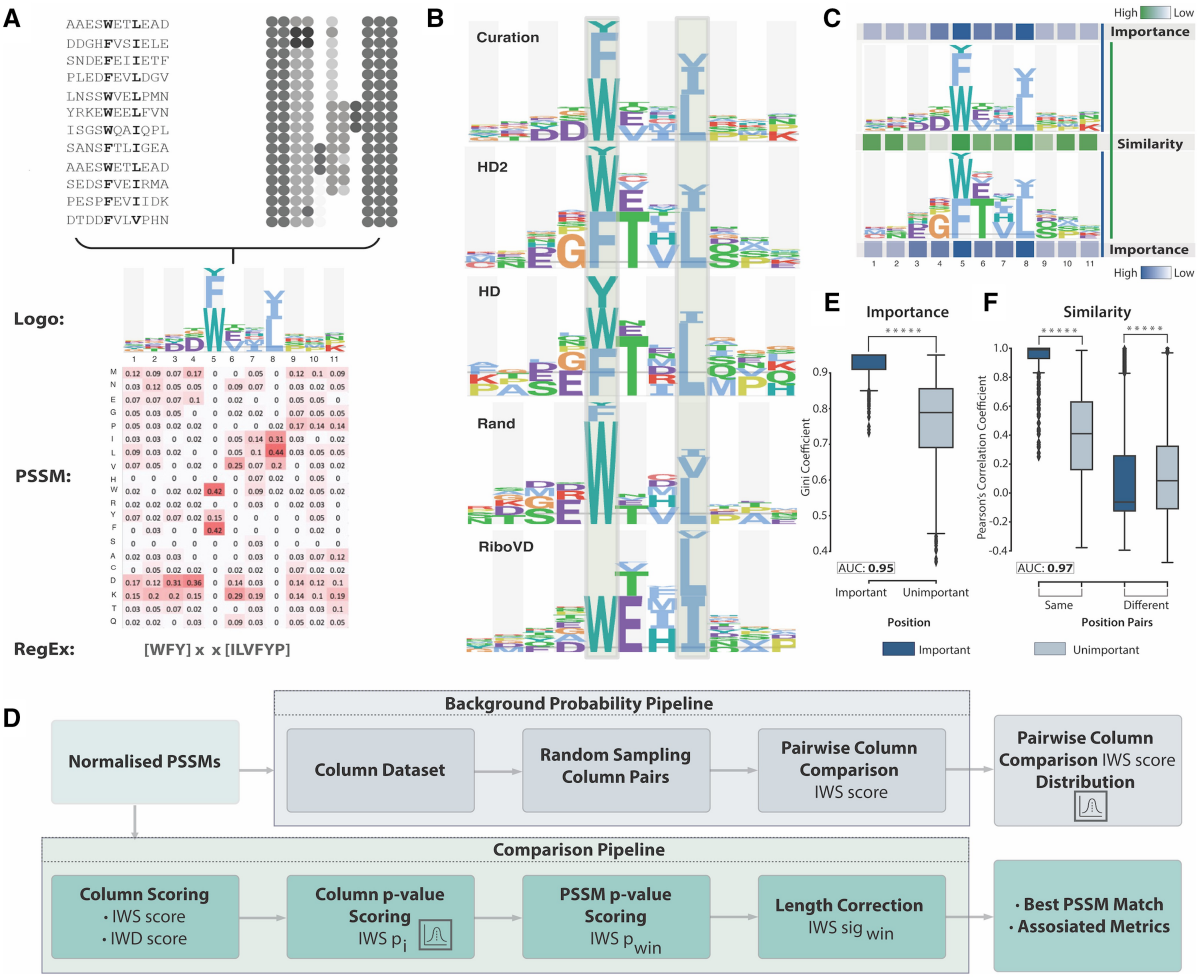
Overview of the CompariPSSM framework. (A) Motif-binding determinants can derive from two major sources of data: inferring the preferences of each residue for each position from a set of motif-containing peptides and direct experimental measurement of amino acid contribution to binding strength (e.g. SPOT arrays or deep mutational scanning). These motif-binding determinants can then be encoded as Logos, Regular Expressions (RegEx) and position-specific scoring matrices (PSSMs). (B) Example of logos of the LIR motif deriving from different sources (curation from ELM, others from ProP-PD screening against distinct libraries). Motif-binding determinants are highlighted by outlined boxes. (C) Schema representing the similar and important positions in motif-binding determinant comparison of two data sources for LIR motifs. For similarity, darker colours denotes more similar positions, and for importance, darker colours indicates more important positions. (D) Schema of the workflow in the CompariPSSM pipeline. The framework includes two major components, one for the PSSM–PSSM comparison and one for defining the background probability of seeing a certain IWS score by chance. The input is two normalised PSSMs and the output is the corrected PSSM–PSSM comparison *p*-value and the PSSM max dissimilarity score. (E) Boxplots of the Gini coefficient for important (fixed, *n* = 1205) and unimportant (wildcard, *n* = 1811) positions in motifs (Mann–Whitney *p*-value <1.0 × 10^−16^). (F) Boxplots of the Pearson’s correlation coefficient for important [*n* = 367 (same); *n* = 4529 (different)], unimportant [*n* = 857 (same); *n* = 10 523 (different)], similar and different positions in motifs. (*****Mann–Whitney *p*-value <1.0 × 10^−16^).

In computational motif discovery, motif-binding determinant models of SLiMs are generally represented as regular expressions (RegEx) or position-specific scoring matrices (PSSMs) (Fig. 1A). RegEx describe the motif consensus as a sequence using single-letter amino acid codes (e.g. ‘A’ for Alanine) to define the important residues, square brackets to define degeneracy (e.g. ‘[KR]’ for any basic residues, or ‘[^KR]’ for no basic residues), ‘^’ for amino termini, ‘$’ for carboxyl termini and dots (‘.’) for undefined positions allowing any residue (Aasland *et al.* 2002). PSSMs represent the motif-binding determinants using a quantitative approach to encode the likelihood of or preference for, an amino acid to occur at a given position in the motif. PSSMs are represented as a matrix, with L columns, where L is the length of the motif peptide and 20 rows, one for each of the standard amino acids. PSSMs store motif-binding determinant models as preference scores for each amino acid at each motif position (Fig. 1B). The PSSM representation has a major advantage over RegEx as it encodes quantitative preferences for the entire peptide sequence and not only qualitative preferences for core amino acids of the consensus.

Motif-binding determinant comparison is a common motif analysis task. Newly discovered SLiM instances can be compared with lists of validated motif classes to check if they belong to a previously characterised class. Similarly, the data from previously characterised classes can be applied to benchmark experimental motif discovery methods such as proteomics phage display and deep mutational scanning. Currently, comparison is performed with the CompariMotif tool (Edwards *et al.* 2008) using RegEx–RegEx comparison; however, there are several issues with this approach. Most notably the requirement to define a RegEx for the motif from the data for comparison and the loss of all quantitative information resulting from this redefinition of the binding determinants. There is a need to develop new approaches which consider quantitative residue preferences of the entire sequence in their comparison. Consequently, we have created a new tool, CompariPSSM, that identifies and scores similarities between motif-binding determinants using PSSM–PSSM comparison.

## 2 Materials and methods

The CompariPSSM framework is a pipeline for the comparison of motif-binding determinants encoded as PSSMs. The framework integrates information on PSSM column similarity, scored using Pearson’s correlation coefficient, and the PSSM column importance, scored using the Gini coefficient, to align the PSSMs and calculate a similarity score for the best alignment (Fig. 1C). CompariPSSM takes as input a query PSSM and a set of one or more comparison PSSMs and outputs the aligned comparison PSSM(s) to the query PSSM with statistical measures of the likelihood of that match happening by chance.

### 2.1 Scoring-binding determinants encoded as PSSM columns

#### 2.1.1 Importance-weighted similarity score

At the core of the CompariPSSM framework is the simple pairwise comparison of two PSSM columns encoding motif-binding determinants (Fig. 1C). CompariPSSM calculates an importance-weighted similarity (IWS) score for each pairwise comparison. The IWS score integrates two key determinants of comparison, the similarity between the two columns and the importance of the two columns. The similarity between two columns in the PSSMs is calculated using Pearson’s correlation, which measures the strength of the linear relationship between two variables. It has a value between −1 and 1, with similarity scores closer to 1 representing more similar binding determinants. Two positions can have high similarity yet encode minimal specificity information, for example, positions where all amino acids are equally likely (e.g. columns 1, 2, 10 and 11 in Fig. 1C). Hence, there is a requirement to weight the similarity score by column importance. The importance of a position in a PSSM describing motif-binding determinants can be defined as the amount of specificity information it encodes, and as such positions in a PSSM with strong outliers in the weights for specific amino acids are more important (e.g. columns 5 and 8 in Fig. 1C), whereas positions in a PSSM that have many residues with similar scores are unlikely to substantially contribute to the specificity and affinity of the motif (e.g. columns 1, 2, 10 and 11 in Fig. 1C). The importance of the position in each PSSM is calculated using the Gini coefficient, a measure of statistical dispersion that calculates the inequality among values. The coefficient ranges between 0 and 1, with values closer to 1 representing more important binding determinants. Using these two metrics, the IWS score of two motif-binding determinants PSSM columns can then be calculated using the following equation:

(1) $$\mathrm{IWS}\;\mathrm{score}=\mathrm{Similarity}{\;A}_{i}B_{i}\times \mathrm{Importance}\;A_{i}\times \mathrm{Importance}\;B_{i}$$

Equation 1: IWS score for PSSM–PSSM column comparison. *A_i_* is column *i* in PSSM_*A*_, *B_i_* is column *i* in the PSSM_*B*_, Similarity *A_i_B_i_* is the Pearson’s correlation between column *A_i_* and column *B_i_*, Importance *A_i_* is the Gini coefficient of column *A_i_*, and Importance *B_i_* is the Gini coefficient of column *B_i_*.

The IWS score encodes, in a simple intuitive manner, the properties required to compare motif-binding determinants. The IWS ranges between −1 and 1, with 1 denoting identical columns with single amino acid-binding determinants (e.g. only proline allowed in the position). Values towards −1 denote no overlap in preferences for highly important positions. Columns must satisfy the requirements for strong-binding determinants in both PSSMs (i.e. both Gini coefficients towards 1) and strong similarity between the binding determinants (i.e. Pearson’s correlation towards 1) to achieve a high IWS score. The IWS score allows single columns to be compared and key-matching-binding determinants to be matched to corresponding positions in other PSSMs. The scoring scheme can be easily expanded to compare two PSSMs of equal length using the following equation:

(2) $${\;\mathrm{IWS}}_{\mathrm{win}}\mathrm{score}=\sum_{i=1}^{n}{\;\mathrm{IWS}}_{i}$$

Equation 2: IWS_win_ score for PSSM–PSSM comparison. IWS_*i*_ is the IWS score for column *i* between two PSSMs of equal length and *n* is the length of the PSSMs.

#### 2.1.2 Importance-weighted dissimilarity score

The IWS score allows shared and important binding determinants to be identified. An additional score that identifies important positions in the query PSSM that are not present in the corresponding position of the comparison PSSM is also important to discriminate between similar but non-identical motif-binding determinants (e.g. proline-rich motifs). Therefore, in parallel to the IWS score calculation, an importance-weighted dissimilarity (IWD) score is calculated. The IWD score is analogous to IWS but looks for the lack of similarity, calculated as the normalised product of the mean absolute error between the columns at a given position in the two PSSMs. As in the IWS score calculation, the IWD score is weighted by the position importance as defined by the Gini coefficient but differs as it only uses the query PSSM importance. The IWD score ranges from 0, denoting that the two positions are identical, to 1 meaning that there is no overlap in the two positions and that the position is required in the query PSSM. The IWD score is calculated using Equation (3). The final PSSM dissimilarity score for the whole PSSM is the maximum dissimilarity score from the position IWD scores. This allows the position most likely to break the binding determinants to be identified.

(3) $$\mathrm{IWD}\;\mathrm{score}=\;\frac{\sum_{a=1}^{20}\left|Q_{i_{a}}-{C_{i_{a}}}_{}\right|}{20}\times \mathrm{Importance}\;Q_{i}$$

Equation 3: IWD score. *Q_i_* is column *i* in the query PSSM, *C_i_* is column *i* in the comparison PSSM, *a* is the iterator for the 20 amino acids in each PSSM at position *i*, and Importance *Q_i_* is the Gini coefficient of column *Q_i_* from the query PSSM.

### 2.2 The CompariPSSM PSSM–PSSM comparison pipeline

The CompariPSSM tool leverages the IWS scoring scheme for pairwise PSSM column comparison to align and score PSSM pairs. The CompariPSSM pipeline includes two major steps (Fig. 1D): (i) the *Background Probability Pipeline* that calculates the likelihood of pairwise PSSM column IWS score using a randomisation-based probabilistic framework and (ii) the Comparison Pipeline which aligns PSSM pairs using a sliding window approach to define the optimal PSSM–PSSM alignment and applies information from the *Background Probability Pipeline* to calculate the likelihood of such a PSSM–PSSM alignment occurring by chance.

#### 2.2.1 Background probability distribution

The *Background Probability Distribution Pipeline* calculates the pairwise column IWS scores distribution by applying a randomisation-based approach (Fig. 1D). This pairwise column IWS score distribution provides the statistical framework for the CompariPSSM PSSM–PSSM comparison pipeline allowing the likelihood of pairwise column comparisons, and therefore pairwise PSSM comparisons, to be quantified. The first step is PSSM min-max normalisation which scales the values to range from 1 to 0 based on the maximum and minimum value of a column in the PSSM to shift the PSSMs to a comparable numeric space (see Supplementary File S1, *PSSM Normalisation*). The next step is the creation of two *PSSM column sets* containing all PSSM columns from the input query or comparison PSSM sets. The complete PSSM column set size influences the accuracy and biases of the statistical framework and as such when possible large PSSM sets should be used. Next, a pair of columns, one each from the query and comparison *PSSM column sets*, are selected randomly and the IWS score is calculated and stored. This step is repeated to build a background distribution of IWS scores (default sampling size of 1 00 000 PSSM column pairs). Once constructed, the IWS score distribution can be used to estimate the probability of seeing a given IWS score by chance in the same dataset.

(4) $${\mathrm{IWS}}_{p}=\mathrm{Dist}(\mathrm{IWS})$$

Equation 4: Importance weighted similarity probability (IWS_*p*_) score for PSSM–PSSM comparison. ${\mathrm{IWS}}_{p}$ is the probability of seeing a given IWS score based on the randomisation-based distribution of IWS scores. The function Dist defines the proportion of randomly sampled PSSM column pairs that score an IWS score greater than or equal to the IWS score of the column pair of interest.

#### 2.2.2 PSSM–PSSM comparison

The *PSSM–PSSM comparison pipeline* step defines the optimal alignment of two PSSMs and scores the likelihood of that alignment. The first step, as in the *Background Probability Distribution Pipeline*, is PSSM min-max normalisation. Next, the comparison is performed by sliding the smaller PSSM across the larger PSSM, one column at a time, and calculating the similarity of each window. In each window, two PSSMs of equal length are compared, and therefore each column from the first PSSM has a corresponding column in the other PSSM. For every corresponding column pair, the IWS and IWD scores are calculated using Equations (1) and (3), respectively. The likelihood of seeing the observed IWS score by chance, IWS_*p*_, is defined based on the distribution of IWS scores of the randomly paired PSSM columns from the *Background Probability Pipeline* as defined in Equation (4). Subsequently, the probability of the comparison window, ${\mathrm{IWS}}_{p_{\mathrm{win}}}$, is calculated as the product of the corresponding pairwise column ${\mathrm{IWS}}_{p_{i}}$ probabilities from the window as follows:

(5) $${\mathrm{IWS}}_{p_{\mathrm{win}}}=\prod_{i=1}^{n}{\mathrm{IWS}}_{p}$$

Equation 5: Importance weighted similarity window probability (${\mathrm{IWS}}_{p_{\mathrm{win}}}$) score for PSSM–PSSM comparison. The ${\mathrm{IWS}}_{p_{\mathrm{win}}}$ is the product of the pairwise column ${\mathrm{IWS}}_{p_{i}}\;$defined on the distribution of the IWS score of the randomly paired PSSM columns from the background probability pipeline, where *n* is the PSSM window length and *i* is the position in the PSSM window.

Finally, the uniform product distribution (UDF) is applied to correct the ${\mathrm{IWS}}_{p_{\mathrm{win}}}$ for the number of columns compared in the window comparison and to define the final window comparison significance score, ${\mathrm{IWS}}_{{\mathrm{sig}}_{\mathrm{win}}}$, as follows:

(6) $${\mathrm{IWS}}_{{\mathrm{sig}}_{\mathrm{win}}}=\mathrm{UDF}({\mathrm{IWS}}_{p_{\mathrm{win}}},\;n)$$

Equation 6: Final PSSM–PSSM comparison probability. The ${\mathrm{IWS}}_{{\mathrm{sig}}_{\mathrm{win}}}$ probability is the final *p*-value for a PSSM–PSSM comparison, and it is defined as the corrected IWS window probability (${\mathrm{IWS}}_{p_{\mathrm{win}}}$) score.

After the sliding window approach has compared all possible PSSM windows for the two PSSMs, the PSSM–PSSM alignment with the most significant ${\mathrm{IWS}}_{{\mathrm{sig}}_{\mathrm{win}}}$ is returned as the optimal alignment, and the ${\mathrm{IWS}}_{{\mathrm{sig}}_{\mathrm{win}}}$ and the maximum IWD score for the window are used as the similarity and dissimilarity scores for the PSSMs (Fig. 1D). The ${\mathrm{IWS}}_{{\mathrm{sig}}_{\mathrm{win}}}$ is then corrected for the number of windows compared by the sliding window approach using Bonferroni correction. If the comparison PSSM set contains more than one PSSM, all PSSMs are compared to the query PSSM and the PSSM with the most significant ${\mathrm{IWS}}_{{\mathrm{sig}}_{\mathrm{win}}}$ is returned as the best matching PSSM. ${\mathrm{IWS}}_{{\mathrm{sig}}_{\mathrm{win}}}$ is also referred to as the final PSSM–PSSM *p*-value of the comparison.

### 2.3 Benchmarking

**Figure 2.**
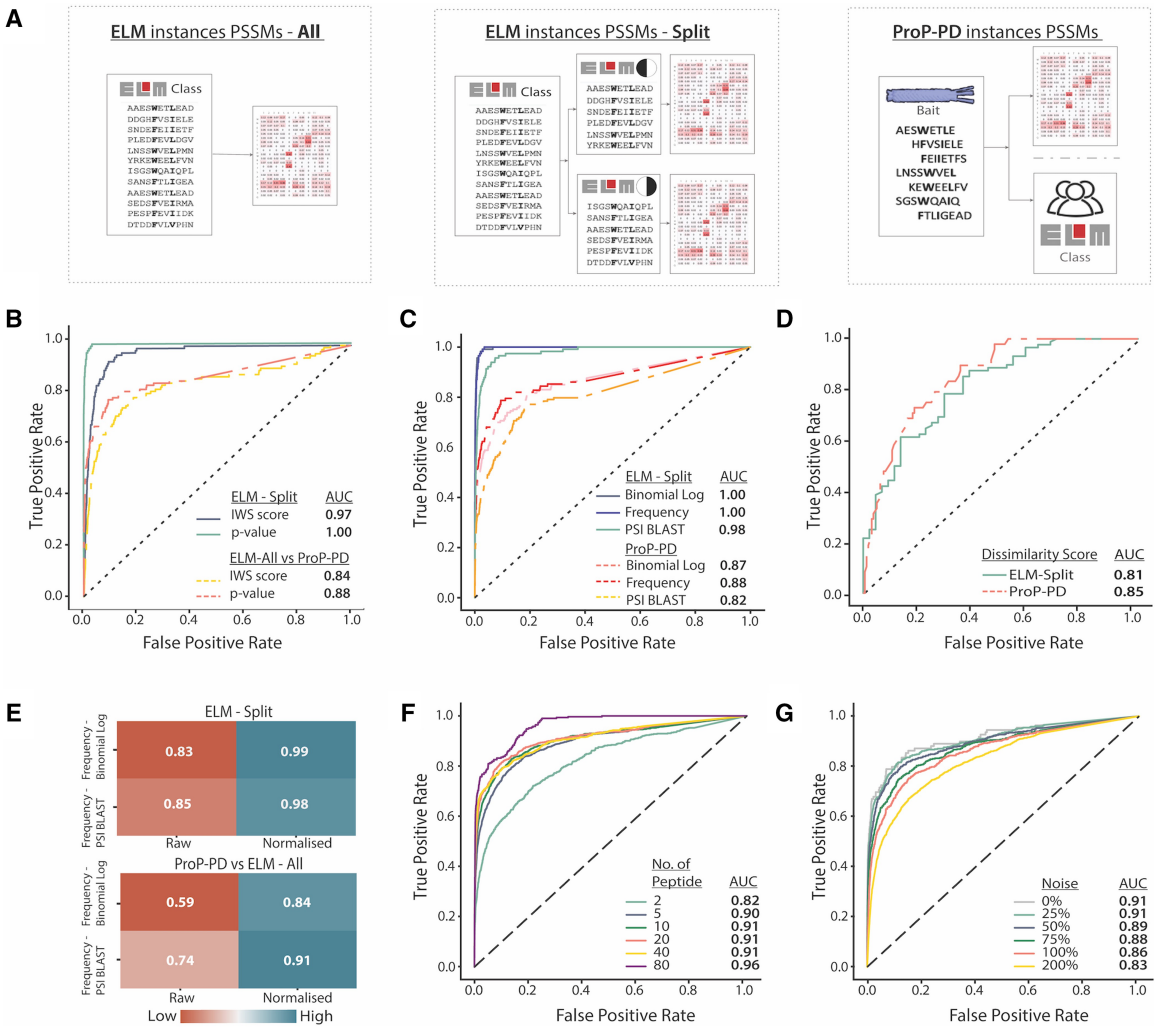
Evaluating CompariPSSM’s ability to identify similar binding determinants. (A) The PSSM datasets used in the CompariPSSM benchmarking. (B) ROC curves comparing the IWS score and *p*-value for their ability to discriminate between motif classes. (C) ROC curves comparing the PSSM construction methods for their ability to identify similar binding determinants. (D) ROC curve of the dissimilarity score between the significant results (*p*-value≤0.001) in the ELM-Split and ELM-All versus Prop-PD datasets. (E) The difference between the AUC of the raw and normalised PSSMs in different datasets. (F) ROC curves of the dataset size sampling based on the ProP-PD datasets. Data sizes range from 2 to 80 peptides. (G) ROC curves quantifying influence of the noise based on the ProP-PD datasets. ProP-PD PSSMs were constructed from 20 peptides with noise added using the following levels: 0% (no noise), 25% (+5 peptides), 50% (+10 peptides), 100% (+20 peptides) and 200% (+40 peptides).

Several PSSM datasets were used to benchmark CompariPSSM. These sets were derived from the ELM database (Kumar *et al.* 2022) and experimental motif discovery methods (Benz *et al.* 2022) (Fig. 2A). A set of manually curated motif-containing peptides from human proteins was also extracted from PDB (Berman *et al.* 2000) to test the CompariPSSM’s ability to annotate motif-containing peptides. Additionally, the AlphaMissense PSSM models for each ELM class were constructed from AlphaMissense scores (Cheng *et al.* 2023) for motif instances containing regions and the conservation PSSM models for each ELM class were derived from protein alignments created using the Gopher algorithm (Davey *et al.* 2007) using *Metazoan* proteomes (Supplementary Table S1) aligned using CLUSTAL Omega (Sievers *et al.* 2011). All components of the CompariPSSM pipeline were benchmarked, specifically the Gini coefficient as an importance metric, Pearson’s correlation coefficient as a similarity metric, the raw score and *p*-value as the algorithm output, and the PSSM scoring and normalisation methods. A detailed description of all the datasets and benchmarking methods can be found in Supplementary File S1.

### 2.4 Availability and implementation

CompariPSSM is available as stand-alone open-source Python software at https://github.com/ifigenia-t/CompariPSSM. The tool is also available as a web server at https://slim.icr.ac.uk/projects/comparipssm. The web server interface is written in JavaScript using the React framework. The server side is written as a Python FastAPI Web Framework. A Kafka management system is used to queue submitted jobs. Results can be downloaded in JSON format. A detailed description of CompariPSSM usage and output is provided on the help page (https://slim.icr.ac.uk/projects/blog?blog_id=comparipssm_help). The CompariPSSM server has been successfully tested on a range of major modern browsers: Safari (v. 15.6.1), Chrome (v. 124) and Firefox (v. 125.0.3).

## 3 Results

### 3.1 Evaluating similarity and importance metrics for motif-binding determinant comparison

#### 3.1.1 The Gini coefficient can discriminate binding determinant importance

The CompariPSSM tool uses Gini coefficient scores to weigh the positions of a binding determinant model by their importance. The power of the Gini coefficient to discriminate between important and unimportant motif positions was tested using the ELM Instances Important Position dataset and the PSSMs of the ELM Instances Dataset—All (Fig. 2A). Important positions were represented by defined consensus positions of the motif (i.e. positions with the one-letter representation of the amino acid or a group of allowed amino acids inside square brackets) and unimportant positions were represented by the non-consensus positions that can be any amino acid (i.e. positions represented as a dot) or prohibited (i.e. defined as [^]). As expected, the Gini coefficient scores for PSSM columns diverged significantly between the important and unimportant motif position groups (Fig. 1E, Mann–Whitney *p*-value ≤ 1.0 × 10^−16^). An area under the curve (AUC = 0.95) analysis verified the Gini coefficient as a strong discriminatory parameter for important binding determinants (Supplementary Fig. S1A). An ‘importance’ cut-off of 0.87 can be defined for the Gini coefficient using Youden’s J statistic (Supplementary Fig. S1A); however, this cut-off is dependent on the PSSM set, necessitating recalculation for each dataset.

#### 3.1.2 Similarity metrics can be applied to binding determinant comparison

The CompariPSSM tool uses Pearson’s correlation coefficient to score the similarity between a pair of columns in a PSSM–PSSM comparison. The Pearson’s correlation coefficient was tested on two sets of column pairs from the *ELM Instances PSSMs Dataset—Split* dataset: the corresponding columns in the two halves of the split PSSMs (*same*), and randomly paired PSSM columns (*different*). As expected, the similarity of the *same* column pair positions was significantly higher than the *different* column pairs for important motif positions (Fig. 1F). Pearson’s correlation coefficient significantly discriminates between the *same* and *different* important positions (AUC = 0.97, Mann–Whitney *p*-value = 5.79 × 10^−198^) (Supplementary Fig. S1B). The same effect was observed for unimportant positions although, with a weaker effect size, this is expected as unimportant positions are free to diverge and therefore are not expected to be highly similar (AUC = 0.74, Mann–Whitney *p*-value = 1.65 × 10^−121^) (Fig. 1F; Supplementary Fig. S1B).

#### 3.1.3 Score quality, PSSM scoring methods and PSSM normalisation

The discriminatory power of the raw PSSM score (IWS score, see Equations (1) and (2) in Section 2) and the PSSM–PSSM *p*-value (the corrected IWS${\mathrm{sig}}_{\mathrm{win}}$, see Equation (6) in Section 2) were tested using the *ELM Instances PSSMs Dataset—Split*, the *ELM Instances Dataset—All* and the proteomic peptide phage display (*ProP-PD*) *dataset* PSSMs (Fig. 2A), with frequency as the PSSM scoring method. The PSSM–PSSM *p*-value showed consistently better discrimination between the matching and non-matching PSSMs compared to the raw PSSM score (Fig. 2B). We also tested two additional PSSM scoring methods (Binomial Log and PSI-BLAST IC) on the two datasets and, unexpectedly, observed that the frequency PSSM scoring outperformed the two more complex PSSM scoring schemes (Fig. 2C). It is noteworthy that although PSI-BLAST IC incorporates an understanding of the physicochemical properties of amino acids, it performs the worst among the three PSSM scoring methods (Fig. 2C). PSI-BLAST methods upweight physicochemically similar residues at a given position. As a result, they can lack a strong discriminatory signal in specific residues, which lowers the similarity with the correct PSSMs while often increasing the similarity with off-target PSSMs. These effects depend on the quality of the binding determinant data encoded in the PSSM, and when limited data is available and the PSSM poorly describes the true binding determinant, intuitively upweighting physicochemically similar residues might enhance predictive power.

Next, the IWD or dissimilarity score was tested. While the PSSM–PSSM *p*-value allows similar PSSMs to be identified, the dissimilarity score allows the difference in *important* positions of the PSSM to be identified. Consequently, the dissimilarity score was tested on only the significant PSSM–PSSM pairs (*p*-value≤0.001). The analysis showed strong discriminatory power for incorrect matches between highly similar PSSMs validating the metric as an effective filtering step for PSSM–PSSM comparison (AUC: ELM-Split 0.81, ProP-PD 0.85) (Fig. 2D). Subsequently, the efficacy of pre-PSSM–PSSM comparison PSSM normalisation was tested. In many applications of PSSM comparison, the source of the PSSM will differ resulting in PSSMs with distinct numerical encoding. We tested the power of PSSM normalisation to improve PSSM–PSSM comparison by comparing *ELM Instances PSSMs Dataset—Split* PSSMs encoded with frequency against PSSMs encoded by Binomial Log and PSI-BLAST PSSM scoring methods. We observed that a min-max matrix normalisation process significantly improves the PSSM–PSSM comparison and allows PSSMs from different sources to be directly comparable with similar accuracy to PSSMs encoded using the same scoring scheme (Fig. 2E; Supplementary Fig. S1C and D).

#### 3.1.4 Impact of motif flexibility and complexity, and peptide noise and dataset size

A major drawback of PSSMs is their inability to encode variable length gaps in motifs. To assess the impact of motif flexibility, we benchmarked CompariPSSM on the ELM Instances PSSMs Dataset—Split, dividing the dataset into two categories: classes with a flexible consensus, which include motifs with variable length segments in the RegEx, and fixed classes without such variability. CompariPSSM showed no difference in its ability to identify matches; ROC curve analysis yielded an identical AUC of 0.99 for both sets (Supplementary Fig. S1E). This consistency may be attributed to the fact that, in our dataset, ELM motifs can be aligned based on their core consensus, which does not include flexible parts, indicating that the core consensus is sufficient for accurate identification. Next, we explored the impact of motif complexity. The ELM Instances PSSMs Dataset—Split was divided into high, medium and low complexity groups based on the probability of the consensus for each class occurring by chance. Benchmarking through ROC analysis revealed good discriminatory power for all groups with the expected improvement as motif complexity increased (AUC low probability: 0.97, medium probability: 1.00, high probability: 1.00) (Supplementary Fig. S1F). Finally, we explored the effect of peptide set size and noise on PSSM quality, and consequently CompariPSSM predictions, using the ProP-PD Instances PSSMs Dataset. Two separate datasets were constructed: the first by randomly selecting defined numbers of peptides from each ProP-PD bait to assess the impact of dataset size, and the second by adding different levels of noise to 20 randomly selected peptides from each ProP-PD bait to evaluate how noise influences comparisons. These datasets were then compared against the ELM Instances PSSMs Dataset using CompariPSSM. As expected, larger peptide sets and lower percentages of noise result in better predictions. However, except in the most extreme cases, even where there is a large proportion of noise or few peptides, CompariPSSM still has strong discriminatory power (Fig. 2F and G). Collectively, these findings suggest that the comparison process is robust.

### 3.2 Benchmarking motif specificity data

A key role of the CompariPSSM tool is the benchmarking of binding determinant data from experimental sources by comparison to expected consensus information.

#### 3.2.1 AlphaMissense motif mutation outcome predictions

To explore the CompariPSSM tool further, we analysed whether PSSMs encoding the predicted mutational effect or evolutionary information reflects the amino acid preferences at each position of a SLiM. The mutation profiles were created using AlphaMissense mutation prediction scores (Cheng *et al.* 2023) which characterise pathogenicity of all single amino acid substitution in the human proteome. We converted the mutation pathogenicity predictions scores for known motif-containing protein regions into AlphaMissense PSSMs (see Supplementary Methods). For the same set of ELM classes, Conservation PSSMs were derived from alignments created by the Gopher algorithm (Davey *et al.* 2007) against the Metazoan proteome set (see Supplementary Methods). Next, for each ELM class the average PSSM were created from the corresponding ELM instance PSSMs (see Supplementary Methods). Finally, the AlphaMissense (233, six motif classes did not have data to produce an AlphaMissense PSSM) and Conservation (239) PSSMs were compared against PSSMs for *ELM Instances Dataset—All*.

**Figure 3.**
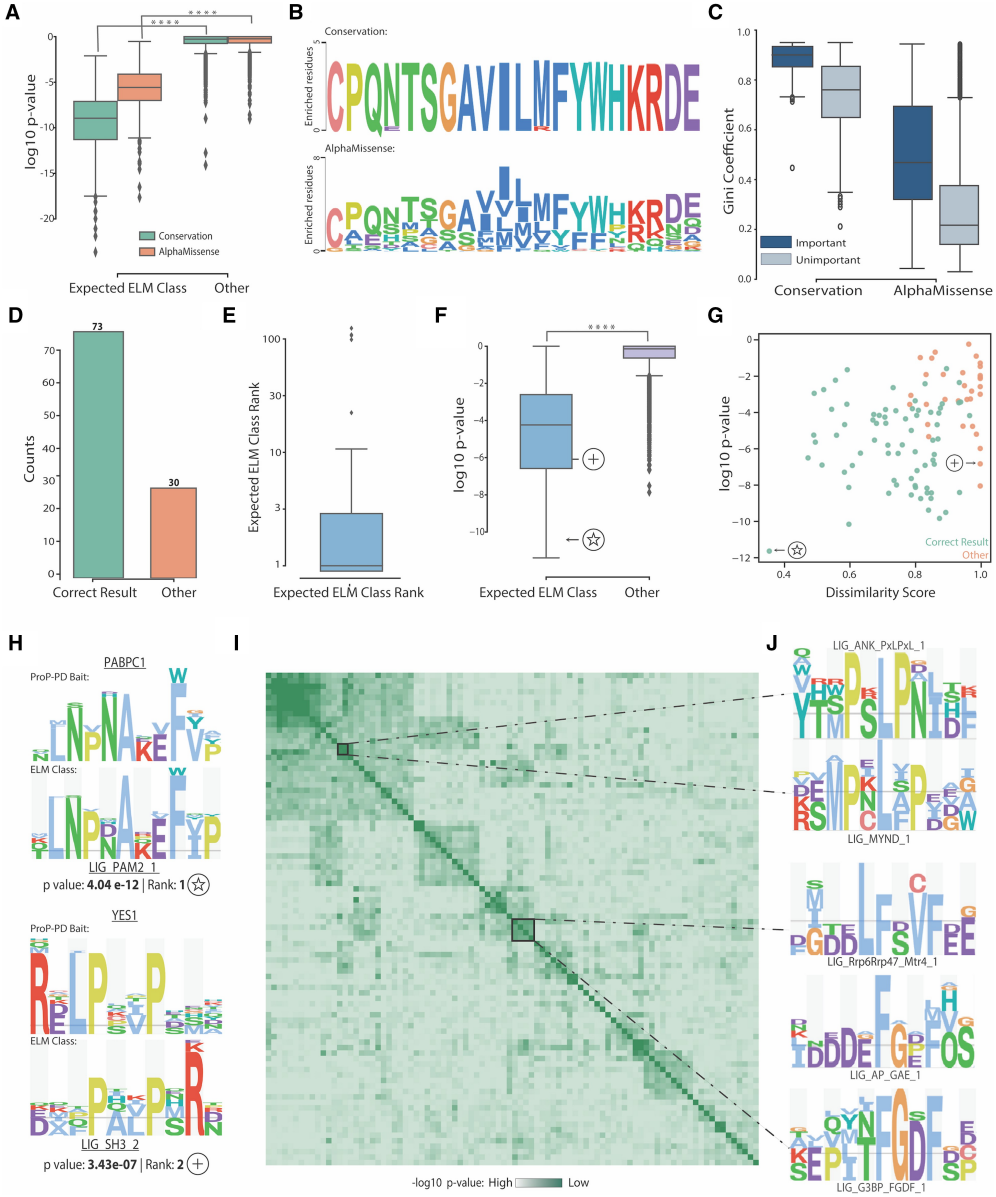
Benchmarking the ProP-PD pilot binding determinants and Similarity of the binding determinants in ELM classes. (A) Boxplot of the *p*-values of expected ELM class against the remaining ELM classes for AlphaMissense (*n* = 233; 67 017) and Conservation (*n* = 237; 68 627) PSSMs. (B) Logos of the fixed positions encoded by the AlphaMissense PSSMs (bottom) and Conservation PSSMs (top). (C) Distribution of the Gini scores for fixed (important *n* = 448) versus other positions (*n* = 1698) encoded in the AlphaMissense and Conservation PSSMs. (D) Numbers of correctly and incorrectly identified ProP-PD baits—ELM class pairs. (E) Distribution of the log_10_ ranking of the expected ELM class (*n* = 103). (F) Boxplots of the *p*-value of the expected ELM class (*n* = 103) against the *p*-value of all the remaining ELM class—ProP-PD bait comparisons (*n* = 21 217) (Mann–Whitney *p*-value <1.0 × 10^−16^). The *p*-values for the comparisons of PABPC1—LIG_PAM2_1 and YES1—LIG_SH3_2 are marked with a star and a plus symbol, respectively. (G) Scatterplot of the log_10_*p*-value of the best-identified matches for each ProP-PD bait and the respective dissimilarity score. In green are reported all the correctly identified pairs as opposed to orange which signifies the incorrect pairs. The *p*-values for the comparisons PABPC1—LIG_PAM2_1 and YES1—LIG_SH3_2 are marked with a star and a plus symbol, respectively. (H) Logos of an example of a correctly identified ProP-PD bait (PABPC1) against ELM class (LIG_PAM2_1) pair and a correctly identified pair (YES1—LIG_SH3_2) with high dissimilarity along with the *p*-value and rank of the comparison, marked with a star and a plus sign, respectively in previous panels. (I) Similarity Matrix of all LIG ELM motif classes. The top left corner denotes a section containing the proline-rich motifs. (J) Logos of ELM classes clustered in panel I.

In total, the correct ELM class was identified as the most similar PSSM for 220 out of 233 AlphaMissense PSSMs with 213 out of the 220 having a significant PSSM–PSSM *p*-value < 0.001 (Fig. 3A; Supplementary Table S2). The conservation PSSMs exhibited increased accuracy, identifying the correct ELM class as the most similar PSSM for 234 out of the 239 tested PSSMs, with 237 out of the 239 of the expected ELM classes showing significance, determined by the PSSM–PSSM *p*-value cut-off of 0.001 (Fig. 3A; Supplementary Table S2). Generally, AlphaMissense and Conservation PSSMs obtained more significant PSSM–PSSM *p*-values for their corresponding expected ELM classes than for all the other compared ELM classes (Fig. 3A, Mann–Whitney *p*-value < 1.0 × 10^−16^ and <1.0 × 10^−16^ for Conservation and AlphaMissense, respectively). However, the AlphaMissense method showed weaker predictive power (Mann–Whitney *p*-value < 1.0 × 10^−16^) than Conservation PSSMs, indicating that the evolutionary PSSMs more robustly encode the core positions of motifs.

We noted that the weaker performance of AlphaMissense PSSMs was often due to physicochemically similar residues permitted in strictly defined important positions. To further investigate this, we analysed the fixed positions of motifs which have been curated by the ELM resource to require a single specific residue at a given position (see Section 2). The columns corresponding to fixed positions in AlphaMissense and Conservation PSSMs were investigated (Fig. 3B; Supplementary Fig. S2A and B) to understand whether they are correctly encoded in AlphaMissense PSSMs. As expected from the initial observations, compared to Conservation PSSMs, AlphaMissense PSSMs allow additional amino acids from physicochemical similar residues in the strict positions that allow only one type of residue (Fig. 3B). To quantify this observation, we calculated the Gini coefficient for important (fixed) and unimportant (wildcard) positions based on the RegEx of the ELM classes. In the case of AlphaMissense, the Gini coefficient values for fixed positions are considerably lower when compared to Conservation, particularly for hydrophobic residues. Given that these residues are strictly required, this suggests that AlphaMissense does not correctly define potentially deleterious residues in SLiMs (Fig. 3C).

#### 3.2.2 Benchmarking ProP-PD motif-binding determinant data

Next, we benchmarked peptide data from a ProP-PD, an *in vitro* protein–peptide interaction discovery method (Davey *et al.* 2017). We applied CompariPSSM to the Benz *et al.* ProP-PD pilot dataset (Benz *et al.* 2022), a screen of known motif-binding domains from the ELM database against a phage library containing 1 million human peptides. For each bait, an ELM class was annotated providing an expected result for the benchmarking comparison (Supplementary Table S3). ProP-PD PSSMs were constructed from peptides aligned by enriched motifs from the results of 103 successful ProP-PD screens and were compared in a pairwise manner by CompariPSSM against 234 binding determinant models from the *ELM Instances Dataset—All* PSSMs (Supplementary Table S4). The CompariPSSM predicted ProP-PD—ELM class pairs were then compared to the expected ELM class to quantify the accuracy of the predictions. CompariPSSM showed that ProP-PD identified the expected ELM class as the top ranked for 73 of 103 screens (recall 71%) and when a significance cut-off of 0.001 is applied, 65 of the screens are correctly identified out of 376 significant hits (recall 63%, precision 17%) (Fig. 3D).

Interestingly, if we filter the significant results to have a dissimilarity score <1, we get 290 significant hits without losing the 65 correctly identified screens (recall 63%, precision 22.4%). CompariPSSM succeeded in ranking the expected ELM class among the top-ranking results in the majority of the cases even for the comparisons where the top result was not the expected result (Fig. 3E) and the *p*-values for the expected ProP-PD—ELM class pairs were significantly higher than other PSSM pairs (Mann–Whitney *p*-value 1.01 × 10^−50^) (Fig. 3F). The binding determinant derived from the ProP-PD screens varied from their expected ELM binding determinant, ranging from nearly identical (e.g. PABPC1) to highly divergent, with several of the incorrectly matched pairs presenting high dissimilarity scores denoting that there is a lack of overlap between the important expected positions (Fig. 3G and H). For example, the ProP-PD determinant screen differed from the ELM database determinant for the YES1 SH3 domain bait as an invariable arginine was expected at the C-terminus of a core PxxP based on the annotated ELM class but observed at the N-terminus in the ProP-PD consensus (Mehrabipour *et al.* 2023) (Fig. 3H). The ProP-PD analysis suggests that CompariPSSM can be applied to specificity determinants from motif analyses to quantify the quality of data produced by experimental motif discovery methods.

### 3.3 Motif-binding determinant clustering

CompariPSSM can also be used to cluster binding determinants by performing multiple pairwise comparisons. An interesting example is the clustering of ELM specificity determinants. As a representative example, took all PSSMs from *ELM Instances Dataset—All* belonging to the ELM ligand binding sites (LIG) motif type and compared them with CompariPSSM calculating the *p*-value for each PSSM–PSSM comparison (Fig. 3I;Supplementary Table S5). A hierarchically clustered heatmap based on a distance matrix defined with the PSSM–PSSM comparison *p*-values revealed several large groups including expected clusters such as a large proline-rich group (Fig. 3I). Several unexpected binding determinant overlaps were also revealed, for instance, the classes LIG_Rrp6Rrp47_Mtr4_1, LIG_AP_GAE_1 and LIG_G3BP_FGDF_1 all share a preference for Phenylalanines (F) two residues apart (Fig. 3J) and, similarly, LIG_MYND_1 and LIG_ANK_PxLPxL_1 contain a shared core PxL motif (Fig. 3J). These results suggest that CompariPSSM has applicability in clustering binding determinants.

### 3.4 Peptide classification

**Figure 4.**
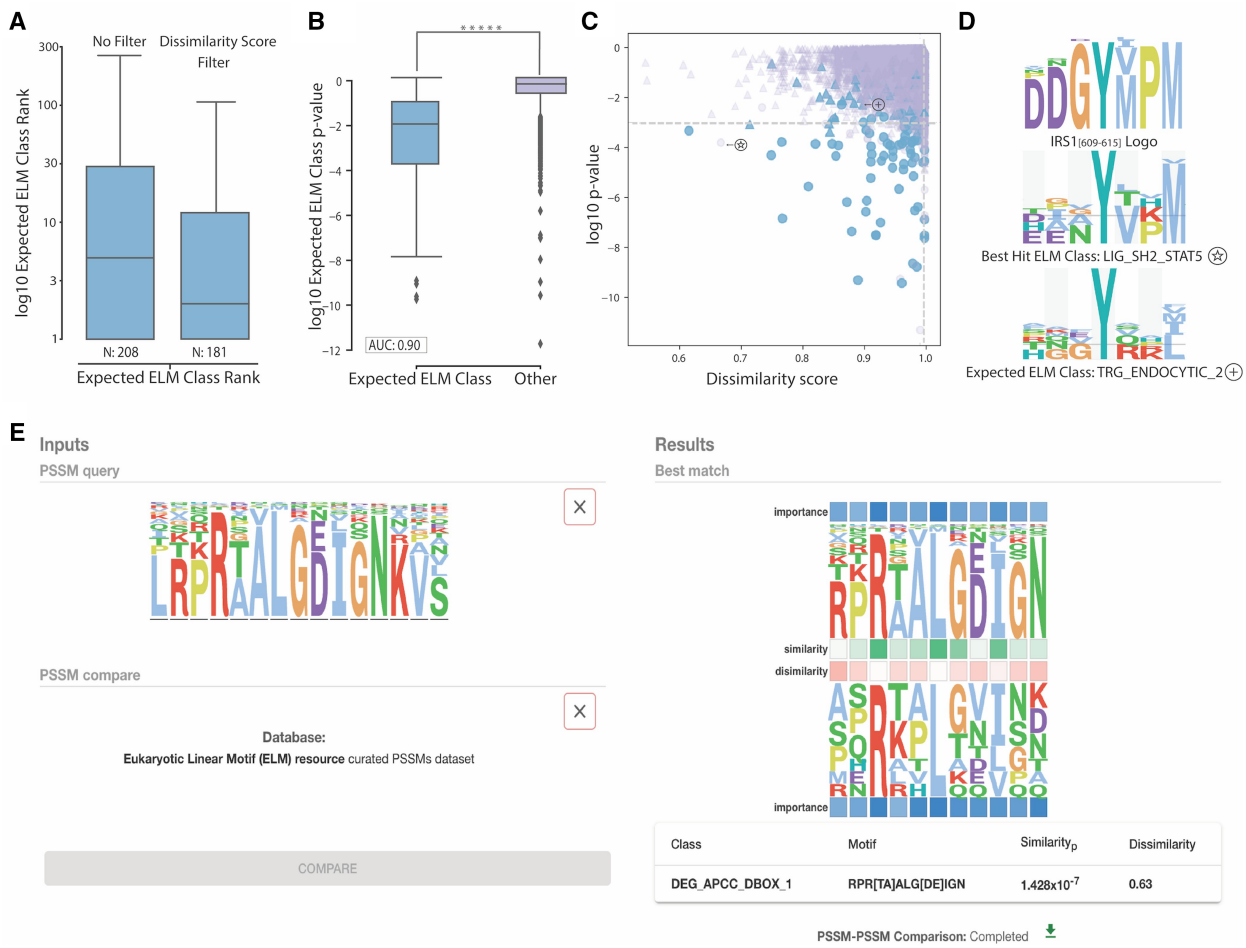
Leveraging evolutionary information for peptide—ELM class classification and the CompariPSSM web page. (A) Distribution of ranking of the expected ELM classes for peptide classification with and without the dissimilarity score filter. (B) Boxplots of the peptide classification PSSM–PSSM *p*-values for the expected ELM classes (*n* = 208) and all the other comparison pairs (*n* = 56 340). Asterisks show the level of significance measured in *p*-values of the Mann–Whitney test (*****p-value <1.0 × 10^−16^). (C) Scatter plot of the log_10_*p*-value and dissimilarity score of all peptide classification comparison pairs. Expected ELM classes are shown in blue as opposed to all other comparisons shown in purple. Results correctly identified are represented as circles, and all the rest are represented as triangles. The star and plus symbols represent the LIG_SH2_STAT5 and TRG_ENDOCYTIC_2, respectively. (D) Motif logo of the human IRS1 [609–615] peptide along with logos of the best match for the LIG_SH2_STAT5 class and the expected ELM class TRG_ENDOCYTIC_2, marked with a star and a plus sign, respectively. (E) Output of the CompariPSSM server, input on left, and results on right. The results show motifs from the query (top) and compare PSSM (bottom) are represented as Logos. The motif class, the motif consensus, the similarity *p*-value and the dissimilarity score are reported as the most significant results of the comparison. Graphical representation of the similarity (in green) and dissimilarity (pink) between two positions and the importance of a position (blue). If there are additional significant results they will also be reported as a list after the best match.

Next, we leveraged evolutionary information for peptide classification by screening peptides against known specificity models of motif-binding pockets using PSSM–PSSM comparison. We used the *Non-ELM Instances Dataset*, a set of orthologue alignment-derived PSSMs from manually curated SLiM instances not present in the ELM database that have been manually classified to a specific ELM class. Among the 208 PSSMs in the dataset, the majority of expected ELM classes were prominently ranked within the top five results. After eliminating peptides with a dissimilarity score of 1, the resulting ranking aligns with most of the expected ELM classes, defining it as the correct outcome (Fig. 4A). Applying a significance cut-off of 0.001 from the 208 peptides, 121 returned at least one significant result and 67 were classified to the correct ELM class (recall: 32.2%, precision: 33.5%; Supplementary Table S6). Filtering to remove all results with a dissimilarity score of 1 resulted in 94 peptides with at least one significant result out of which 63 were correctly identified (recall: 30.3%, precision: 48.8%).

There was a significant difference between the PSSM–PSSM comparison *p*-values of the correctly and incorrectly classified peptides (Fig. 4B, Mann–Whitney *p*-value <1 × 10^−16^) and the ROC analysis shows that the *p*-value strongly discriminates ELM class classification (AUC: 0.90, Supplementary Fig. S2C). Upon further investigation, many of the incorrectly classified motifs were in fact highly similar PSSM pairs, as an example, a PSSM built from an alignment of a region from insulin receptor substrate 1 (IRS1) [609–615] (Choi *et al.* 2019) (Fig. 4D, top), expected to match the TRG_ENDOCYTIC_2 ELM class, returned LIG_SH2_STAT5 class as the best match (*p*-value: 0.00017, dissimilarity score: 0.66) (Figs 3C and 4D middle). The expected ELM class, TRG_ENDOCYTIC_2, a tyrosine-based endocytic sorting signal responsible for the interaction with the Mu subunit of adaptor protein (AP) complex was ranked fifth (*p*-value: 0.0109, dissimilarity score: 0.93) (Figs 3C and 4D bottom). Both TRG_ENDOCYTIC_2 and LIG_SH2_STAT5 are tyrosine-based motifs and visual inspection of the logos of the peptide and ELM classes (Fig. 4D) suggests that the IRS1 peptide is more similar to the STAT5 Src Homology 2 (SH2) domain binding motif than endocytic sorting signal. The three sets of binding determinants share the core central tyrosine; however, valine, proline and methionine enriched in positions five, six and seven of the motifs are stronger determinants in the LIG_SH2_STAT5 and IRS1 PSSMs. From these results, we can conclude that CompariPSSM can be used to classify protein regions using evolutionary information to motif class specificity models.

### 3.5 CompariPSSM web server

The CompariPSSM pipeline and interactive visualisations have been made available as a web server at https://slim.icr.ac.uk/projects/comparipssm. The CompariPSSM server has numerous input options: (i) input PSSMs, which can be copied and pasted directly in or upload a PSSM in a Tab-Delimited Table or JSON format; (ii) sets of aligned or unaligned peptides [in the case of unaligned peptides, the peptides are aligned with the FaSTPACE peptide alignment software v.1.0.1 (https://pypi.org/project/fastpace/) (Kotb and Davey 2024) and the PSSM is generated based on the returned alignment] and (iii) protein regions defined by UniProt accession, gene name or protein name, and region and start and stop offsets. The query PSSM can be compared against a user-defined input PSSM or the eukaryotic linear motif (ELM) resource-curated PSSM dataset (Fig. 4E). The output is the best match to the query PSSM, the comparison similarity (${\mathrm{IWS}}_{{\mathrm{sig}}_{\mathrm{win}}}$) and dissimilarity score (max IWD score), and logos visualising the PSSM–PSSM comparison. If there is more than one significant match, additional hits are shown in a tabular format (Fig. 4E).

## 4 Conclusion

Motif-binding determinant models are key data representation formats that underlie motif analysis. These models are commonly used in motif studies and can be applied to a range of tasks such as motif annotation, classification, clustering and discovery, and can also be employed to benchmark motif discovery methods. Here, we presented CompariPSSM, a new tool for motif-binding determinant comparison, that quantifies the similarity between motif-binding determinants using sliding window PSSM–PSSM comparison and scores PSSM similarity with a randomisation-based probabilistic framework. CompariPSSM complements the functionality of our previous work on CompariMotif (Edwards *et al.* 2008), a tool to identify similarities between motif consensuses by comparing their RegEx. CompariPSSM provides two metrics, a similarity score to quantify whether two specificity determinant models are more similar than expected by chance, and a dissimilarity score that identifies specificity determinants that differ in important positions in the query PSSM (Fig. 3H). Both metrics are necessary to understand if two PSSMs are describing the same specificity determinants. The similarity score is the more important metric, and a significant similarity score signifies that amino preferences encoded by the PSSMs are similar across several positions. Dissimilarity scores flag cases where key positions in the query PSSM are not present in the comparison PSSM. Importantly, the interpretation of dissimilarity scores depends upon the use case. When well-defined specificity determinant models are used, the lack of a key residue is likely to mean that the two models do not describe the same motif class. However, in other cases different interpretations are possible, for example, with poorly defined PSSMs—due, for example, to a limited number of peptides for PSSM construction—the dissimilarity can be interpreted more leniently.

The CompariPSSM tool can be applied across a range of common SLiM analysis tasks. Firstly, it can be used to benchmark the results of experimental motif discovery methods, such as motif deep mutational scanning or high-throughput peptide binding assays, by comparing the newly characterised specificity determinants to expected determinants from previous data. Here, the tool was applied to experimental ProP-PD screens allowing the quality of the ProP-PD selections to be benchmarked. Also, it was applied to benchmark the motif-binding determinants derived from computational approaches based on the AlphaMissense and Conservation information allowing the quality of both methods to be benchmarked. Secondly, PSSM–PSSM comparison can be used for peptide clustering to define overlapping binding determinants, as presented here in an analysis of the ELM motif classes. Additionally, it can be used for annotation of putatively functional disordered regions by comparison to previously characterised motif-binding determinants thereby assigning function to these protein regions. Here, we used CompariPSSM to annotate motif-containing peptides deriving from the PDB database (Berman *et al.* 2000) that were not already in the ELM database and succeeded in correctly classifying half of them with high precision.

A notable limitation of PSSMs is their inability to accommodate variable length gaps or encode interdependencies between positions. These issues could be addressed by employing more complex models such as hidden Markov models (HMMs) or stochastic context-free grammars (SCFGs) instead of PSSMs. HMMs and SCFGs can describe a wide range of SLiM structures, including those with complex patterns and dependencies. These methods not only capture positional amino acid preferences but also characterise gap patterns in peptide alignments, enabling searches with gapped alignments. However, HMMs require a considerable amount of training data to accurately estimate model parameters, which can be a significant hurdle given the limited availability of high-quality data in biological contexts. SCFGs, while powerful for modelling complex, hierarchical dependencies, are computationally demanding and complex to implement, often requiring sophisticated algorithms and extensive computational expertise. Furthermore, both approaches are difficult to visualise and are therefore inherently less intuitive. These challenges make both HMMs and SCFGs less practical for routine use in motif comparison and discovery, despite their potential advantages.

The presence of bias in the datasets used for PSSM comparison is another critical aspect that warrants consideration. One prevalent form of bias stems from the tendency to recognise sequences that conform to established consensus motifs. This bias is then reflected in how experiments are designed resulting in analyses that inadvertently favour the detection and validation of motif instances conforming to existing consensus sequences. Consequently, the dataset used to construct PSSMs are often skewed towards motif consensuses that are already well established in the literature, potentially overlooking less conventional or divergent binding preferences. However, it is worth noting that novel high-throughput experimental methods, such as ProP-PD (Davey *et al.* 2022), may be less susceptible to bias due to the scale of their peptide libraries. These methods, which do not rely on prior knowledge of established motifs, have the potential to yield datasets with a more diverse representation of peptides for a given motif family (Davey *et al.* 2023). As a result, the quality of PSSMs derived from such unbiased datasets is likely to be higher, as they encompass a broader range of sequence variations and may capture novel binding determinants that have been overlooked in biased datasets. Moving forward, efforts to mitigate bias in motif analysis are imperative for enhancing the accuracy and comprehensiveness of motif analyses. Leveraging unbiased experimental approaches and actively seeking out divergent motifs can contribute to the development of models that more accurately reflect the motif-binding determinants for a given pocket.

In conclusion, we have created CompariPSSM, a novel tool for PSSM–PSSM comparison that fills a gap in the current motif analysis tool space. The tool was tested on motif specificity determinant information from the ELM motif resource and ProP-PD screens and proved its applicability across a range of motif analysis tasks. We expect CompariPSSM to be a useful new tool for the motif biology community in their continued characterisation of this underexplored corner of the interactome.

## Supplementary Material

btae644_Supplementary_Data

## Data Availability

CompariPSSM is implemented as an open-source Python tool. The source code is available on GitHub at https://github.com/ifigenia-t/CompariPSSM. Additionally, the CompariPSSM tool is accessible as a web server for public use at https://slim.icr.ac.uk/projects/comparipssm.
