# Supplementary material for: CompariPSSM: a PSSM–PSSM comparison tool for motif-binding determinant analysis
Source: Bioinformatics. 2024 Oct 29;40(11):btae644. doi: 10.1093/bioinformatics/btae644 (PMC11639669; doi:10.1093/bioinformatics/btae644)
Supplement: btae644_Supplementary_Data [file btae644_supplementary_data.zip › CompariPSSM - Supplementary File 1 - Resubmission.docx]

**CompariPSSM: a** **PSSM-PSSM comparison tool for motif binding determinant analysis** **- Supplementary Figures and Material**

**Supplementary Figures**


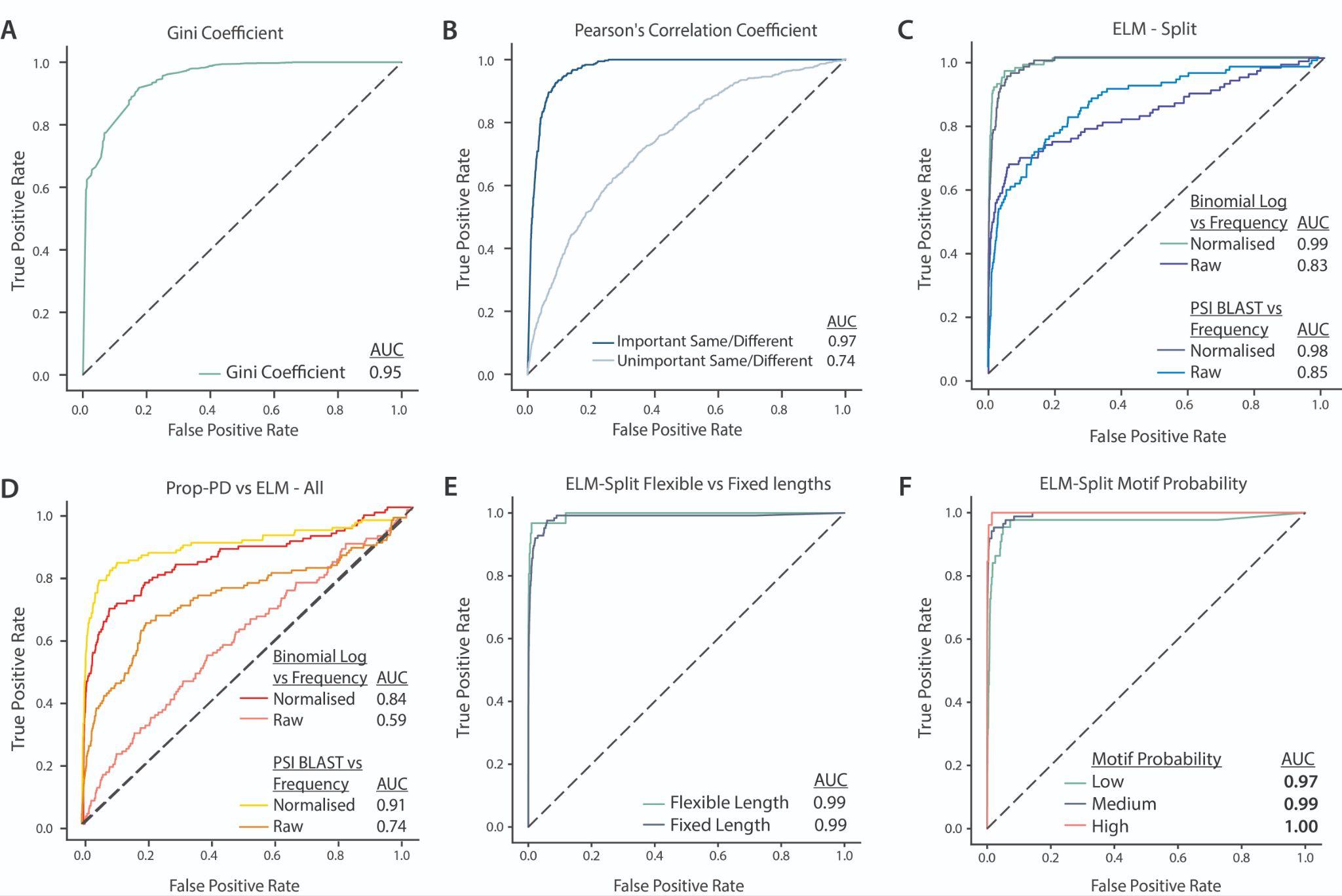
**Supplementary Figure 1.** ROC curve analysis of (A) the Gini Coefficient between Important (TP) and unimportant (FP) positions. (B) Pearson’s Correlation Coefficient (between groups Important/Same - Important/Different positions in blue and Unimportant/Same -'Unimportant/Different positions in grey). (C) ROC curves of the raw and normalised PSSMs constructed with different methods for the ELM-Split dataset and (D) the ProP-PD vs ELM-All set. (E) ROC curves for the flexible and fixed length ELM classes, using the ELM-Split dataset. (F) ROC curves of the ELM classes split based on the probability of the consensus for each class occurring by chance (low probability > 0.001, medium probability: between 0.001 and 0.00001, high probability < 0.00001).


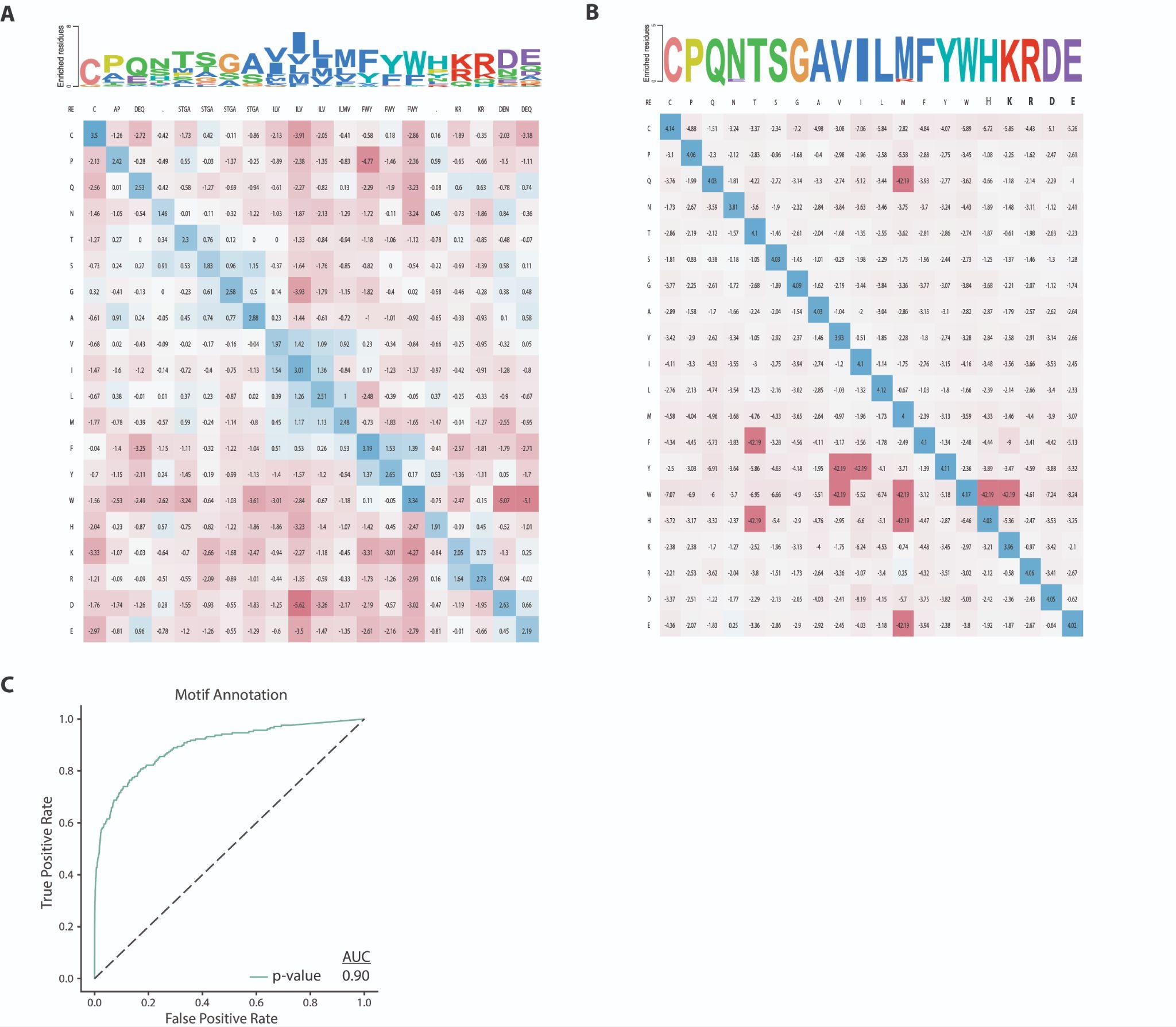


### **Supplementary Figure 2.** (A, B) Logo and heatmap of defined positions for AlphaMissense (A) and Conservation (B) PSSMs. The heatmap represents the log2 of average scores of PSSM columns across all ELM-defined positions. The columns represent an expected fixed amino acid in the following order: CPQNTSGAVILMFYWHKRDE. (C) ROC curve illustrating the discrimination between the comparison p-values for correctly and incorrectly classified motif-containing peptides.

###

**Supplementary Material**

### **Benchmarking datasets**

CompariPSSM has been benchmarked on three sources of motif class binding determinant information encoded as peptide alignments of experimentally validated motif instances (**Figure 2A**): (i) the Eukaryotic Linear Motif (ELM) database, a database of manually curated SLiMs categorised into motif class by motif-binding pocket [(1)](https://sciwheel.com/work/citation?ids=12175714&pre=&suf=&sa=0), (ii) Proteomics Peptide Phage Display (ProP-PD) screens from [(2)](https://sciwheel.com/work/citation?ids=12301587&pre=&suf=&sa=0), an *in vitro* peptide binding assay for the discovery of motif-mediated interactions in intrinsically disordered regions of the human proteome; and (iii) a set of manually curated human SLiMs. The ELM dataset consists of a manually curated set of classified instances that represent the ground truth of the binding determinants of a given motif class. The ProP-PD data results from high throughput screening against domains that are representative of an ELM motif class and these peptide instances include experimental noise.

### ELM dataset

276 ELM classes obtained from the Eukaryotic Linear Motif resource were used to benchmark CompariPSSM. Distinct ELM class subsets were used for distinct parts of the benchmarking depending on the analysis requirements. Supplementary Table 7 provides details on which information was used in each benchmarking analyses:

- *CompariPSSM benchmarking - PSSM construction benchmarking* - 234 ELM classes with at least 2 ELM instances.
- *CompariPSSM benchmarking* - *Important position metri*cs - 202 ELM classes with at least 2 ELM instances. We excluded modification motifs (i.e. MOD) from this set.
- *CompariPSSM benchmarking* - *Ligands clustering* - 94 ELM ligand classes (LIG) with more than 3 ELM instances were used.
- *ProP-PD benchmarking* - 48 ELM classes mapped on ProP-PD baits.
- *AlphaMissense PSSMs benchmarking* - 233 ELM classes with at least 2 ELM instances. This dataset includes only ELM classes with human instances as AlphaMissense mutation predictions scores are available only for the human proteome.
- *Conservation PSSMs benchmarking* - 239 ELM classes with at least 2 ELM instances. This dataset is also limited to human ELM classes to compare the results with AlphaMissense PSSMs benchmarking.
- AlphaMissense and Conservation PSSMs fixed positions - 201 ELM classes. This dataset included ELM classes which had at least one single amino acid requirement at specific position (not flexible part) derived from ELM consensus, and had information from AlphaMissense mutation prediction scores and Metazoa alignments.

*ELM class curated consensus and alignments*

ELM-derived class consensus was simplified to represent the core of the motif and was derived from the PSSMSearch framework based on the aligned the ELM instances.

ELM instance alignments

First, we aligned ELM instances based on the simplified version of ELM consensus which replaced the flexible flanking regions and prohibited positions with wildcard positions. N- or C-terminal wildcard positions were removed. Next, each simplified ELM consensus was extended with surrounding two wildcard positions (excluding N- or C-termini motifs which were extended only on one side). In case of a very short motif (up to length equal to 5), the consensus was first extended with wildcard positions. For example, for LIG_Integrin_RGD_1 represented as ‘RGD’ we used instances matching ‘.RGD.’. The resulting simplified ELM patterns were used to align the motif instances for each ELM class.

ELM class curated consensus

*ELM class curated consensus* was obtained from aligned ELM instances for each ELM class. The aligned instances were submitted to PSSMSearch to obtain the PSSM and the enriched motif. The returned motif does not contain prohibited or flexible representations and it defines the enriched positions based on binomial statistics and rules described in the PSSMSearch paper. We used this representation of ELM motifs to analyse the important and unimportant positions which match the PSSM length. The

ELM class curated motifs can be found in Supplementary Table 7.

ELM peptide sets

These data have been used to create four benchmarking datasets:

- *ELM Instances PSSMs - All Dataset* was created from motif instances from the ELM database. For each ELM class, peptides were extracted and aligned using the simplified ELM class consensus (see above for details).
- *ELM Instances PSSMs - Split Dataset*: Based on the *ELM Instances Dataset - All* peptides, for each ELM class, the peptides were split into two sets of approximately equal size, and each peptide set was aligned using the ELM class consensus (see above for details).
- *ProP-PD Instances PSSMs Dataset* was created from peptides resulting from the *Benz et al* ProP-PD analysis [(2)](https://sciwheel.com/work/citation?ids=12301587&pre=&suf=&sa=0). The analysis screened 40 known motif-binding proteins from the ELM database against several libraries and sub-libraries encoding 16-mer peptides from the human proteome resulting in 227 selections. The selected ProP-PD peptides from each phage display selection were analysed using the SLiMFinder motif consensus discovery method [(3)](https://sciwheel.com/work/citation?ids=5486474&pre=&suf=&sa=0) and 103 selections had an enriched consensus in the selected peptides [(2)](https://sciwheel.com/work/citation?ids=12301587&pre=&suf=&sa=0). A peptide alignment was created for each of the 103 selections by aligning the phage-selected peptides around the enriched SLiMFinder consensus. The expected ELM class was curated for 103 selections, where more than 4 peptides were present to create a PSSM, to create the *ProP-PD - Expected ELM Class* dataset (**Supplementary Table 2**).
- *Non-ELM Instances PSSMs Dataset* was created from a set of 208 manually curated SLiMs that are not currently annotated in the ELM database but can be manually classified to an ELM class, to define the expected results. Orthologue alignments were produced for the proteins containing the peptides using the GOPHER algorithm against a database of metazoan proteomes (**Supplementary Table 1**) [(4)](https://sciwheel.com/work/citation?ids=2637617&pre=&suf=&sa=0).

### Benchmarking PSSM construction

Three methods (i.e., Frequency, Binomial Log and PSI-BLAST) for PSSM construction were used in the CompariPSSM benchmarking for the following datasets: *ELM Instances PSSM Split, ELM Instances - All, ProP-PD Dataset* and *Non-ELM Instance*s. The three methods were chosen as they encoded the specificity data in distinct ways allowing the normalisation approaches to be compared on PSSMs with unique properties encoding the same data. PSSMs were calculated from peptide alignments for all three PSSM construction methods using the PSSMSearch web application with default parameters [(5)](https://sciwheel.com/work/citation?ids=5388579&pre=&suf=&sa=0). The three methods were: (i) the simple *Frequency* scoring method which encodes the amino acid frequencies at each position in the alignment, (ii) the *Binomial Log* scoring method which encodes the over- or under-representation of each amino acid at each position in the alignment using the log of the binomial cumulative function thereby introducing negative values to the comparison and encoding the frequency relative to a background amino acid frequency expectation, and (iii) the PSI-BLAST scoring method, which is an adapted version of the PSI-BLAST algorithm with corrected background amino acid frequencies [(6)](https://sciwheel.com/work/citation?ids=27750&pre=&suf=&sa=0) that introduces pseudo counts to upweights amino acid scores based on their similarity to frequent amino acids in the alignment thereby introducing biologically relevant noise into the benchmarking PSSM.

### Protein Alignment PSSMs

An orthologue alignment was created for each protein by running the GOPHER algorithm [(4)](https://sciwheel.com/work/citation?ids=2637617&pre=&suf=&sa=0) against a search database of metazoan proteomes retrieved from UniProt (**Supplementary Table 1**). PSSMs were constructed from the peptide alignment using frequency scoring.

## PSSM Normalisation

The default input of CompariPSSM is two PSSMs. The first step in the comparison algorithm is the normalisation of the PSSMs to make sure that PSSMs constructed with different methods are comparable to each other. All PSSMs are normalised before the comparison using the following equation:

$norm x_{ij}=\frac{x_{ij} - min(x)}{max(x) - min(x)}$

***Sup. Equation 1:*** ***PSSM Normalisation.*** *Where* $x_{ij}$ *is the binding determinant value for column i and row j of the PSSM, min(x) and max(x) are the minimum and maximum values in the PSSM.*

## CompariPSSM Benchmarking

### The Gini Coefficient as an Importance Metric

The Gini Coefficient was benchmarked as an importance metric on the *ELM Instances Dataset - All* alignments converted to *Frequency* PSSMs*.* The *ELM Instances Important Position* dataset of important and unimportant positions in each ELM class was constructed using the PSSM-derived consensus for each class (e.g., the KEAP1 degron consensus [DNS].[DES][TNS]GE, see section *ELM class curated consensus*). Important positions were represented by defined consensus positions of the motif (i.e., positions with the one-letter representation of the amino acid or a group of allowed amino acids inside square brackets) and unimportant positions were represented by the non-consensus positions that can be any amino acid (i.e., positions represented as a dot). The PSSM-derived consensus motifs do not contain prohibited positions. Columns in the *ELM Instances Dataset - All* PSSMs were defined as unimportant or important based on the definition above and Gini coefficients were calculated for each position. The distributions of the Gini coefficients for the unimportant or important sets were plotted as boxplots and the discriminatory ability of the Gini coefficient was quantified using a Receiver Operating Characteristic (ROC) analysis and the Mann-Whitney U rank test.

### Pearson's Correlation Coefficient as a similarity metric

The Pearson's Correlation Coefficient was benchmarked as a similarity metric on the *ELM Instances PSSMs - Split* dataset converted to *Frequency* PSSMs. For this purpose, every column pair in all PSSM train-test pairs for a specific ELM class was compared and Pearson's Correlation Coefficient was calculated for each comparison. Important and unimportant positions, and therefore columns, were defined as described in the Gini Coefficient benchmarking section. The column pairs were then classified as follows: *Important - Same* for two columns for the corresponding important position in the train-test pairs, *Unimportant - Same* for two columns for the corresponding unimportant position in the train-test pairs, *Important - Not Same* for two columns for important but randomly selected train-test pairs, *Unimportant - Not Same* for two columns for unimportant and but randomly selected train-test pairs. The distributions of the Pearson's Correlation Coefficient for the four sets were plotted as boxplots and the discriminatory ability of the Pearson's Correlation Coefficient was quantified using a Receiver Operating Characteristic (ROC) analysis and the Mann-Whitney U rank test.

### Raw Comparison score vs. p-value

The Importance Weighted Similarity raw score, ${IWS}_{win}score$, and the Importance Weighted Similarity significance score, ${IWS}_{{sig}_{win}}$, were compared on the ELM PSSMs from the *ELM Instances PSSMs Dataset - Split* for their ability to correctly match the train split PSSM to the test split PSSM. The raw score and significance were calculated for each train split PSSM against all test splits in the dataset. The discriminatory ability of each metric was quantified using a Receiver Operating Characteristic (ROC) analysis. The same analysis was repeated with the *ProP-PD PSSMs Dataset* compared against the *ELM Instances PSSMs Dataset - All.*

### PSSM Scoring Methods

The contribution of the PSSM creation method to PSSM comparison quality was benchmarked on the *ELM Instances Dataset - Split, - All and ProP-PD Dataset*. For *ELM Instances PSSMs Dataset - Split*, the PSSMs created from the query split ELM class were compared against PSSMs created from the comparison splits ELM classes using the Frequency, Binomial Log and PSI-BLAST method. For the *ProP-PD Dataset*, the PSSMs created from the *ELM Instances PSSMs Dataset - All* alignments were compared against PSSMs created from the *ProP-PD Dataset* alignments using the Frequency, Binomial Log and PSI-BLAST method. The discriminatory ability of each metric was quantified using a Receiver Operating Characteristic (ROC) analysis.

### PSSM Normalisation

The contribution of PSSM normalisation to PSSM comparison quality was benchmarked on the *ELM Instances Dataset - Split, - All and ProP-PD Dataset*. PSSMs were created for each dataset using the Frequency, Binomial Log and PSI-BLAST PSSM scoring methods. For *ELM Instances PSSMs Dataset - Split*, the PSSMs created from the train alignments with the Frequency method were compared against PSSMs created from the train alignments using the Binomial Log and PSI-BLAST method with and without normalisation. For the *ProP-PD Dataset*, the PSSMs created from the *ELM Instances Dataset - All* alignments with the Frequency method were compared against PSSMs created from the *ProP-PD Dataset* alignments using the Binomial Log and PSI-BLAST method with and without normalisation.

### Dataset Size

The *ProP-PD Instances PSSMs Dataset* was used to benchmark the effect of dataset size. Peptides were randomly selected to create sets of size 2, 5, 10, 20, 40, and 80 for each ProP-PD selection, and frequency PSSMs were created for each set. This process was repeated five times for each ProP-PD bait, resulting in 2143 frequency PSSMs, which were then benchmarked against the *ELM Instances PSSMs Dataset - All* using CompariPSSM. The obtained results were quantified with ROC curves.

*Noise Sampling*

The *ProP-PD Instances PSSMs Dataset* was used to benchmark the effect of noise on the PSSM-PSSM comparison. First, for each ProP-PD selection with at least 20 peptides (70 selections) we created peptide alignments from 20 randomly selected peptides to fix the motif-containing peptide sample size when adding the noise. Next, we added randomly selected peptides from other Pro-PD baits to introduce noise. The noise sizes were as follows: 5 (25%), 10 (50%), 15 (75%), 20 (100%) and 40 (200%) peptides. This process was repeated five times for each ProP-PD bait. The noise peptides were adjusted to match the length of the reference bait alignment. Finally, these peptide alignments were used to create frequency PSSMs, resulting in 1,700 frequency PSSMs, which were run against *ELM Instances PSSMs Dataset- All* using CompariPSSM. The obtained results were quantified with ROC curves.

### Motif Flexibility

The *ELM Instances PSSMs Dataset - Split* was used to benchmark the effect of motif flexibility on the PSSM-PSSM comparison. The ELM-defined consensus was used to divide the ELM dataset into classes with fixed and flexible consensus. The flexible ELM classes had motif motif consensuses that encode flexible parts in the regular expression that could change the length of the matching motif (e.g., D(S)G.{2,3}([ST]) for DEG_SCF_TRCP1_1). The fixed classes had motif consensuses without such variable length elements. For each set, flexible and fixed, CompariPSSM was benchmarked on the ability to correctly match the train split PSSM to the test split PSSM (see *ELM Instances PSSMs Dataset - Split)*. The results were quantified with ROC curves. The flexible motif classes can be found in the Supplementary Table 7.

### Motif Complexity

The *ELM Instances PSSMs Dataset - Split* was used to benchmark the effect of motif complexity on the PSSM-PSSM comparison. The motif probability indicates the likelihood of encountering a given motif in any protein sequence by chance. A lower motif probability suggests that the ELM class consensus has more positions with defined preferences, or positions with strict preferences without degeneracy. Based on the ELM motif probability provided for each ELM class by the ELM resource, we classified the ELM classes into three categories: high-quality (motif probability < 0.00001), medium-quality (motif probability between 0.001 and 0.00001), and low-quality (motif probability > 0.001). For each category, CompariPSSM was benchmarked on the ability to correctly match the train split PSSM to the test split PSSM (see *ELM Instances PSSMs Dataset - Split)*. The results were quantified with ROC curves. The ELM motif probability can be found in the Supplementary Table 7.

## AlphaMissense PSSMs Benchmarking

The AlphaMissense prediction scores [(7)](https://sciwheel.com/work/citation?ids=15380065&pre=&suf=&sa=0) characterise mutation pathogenicity and are available for all amino acid substitutions in the human proteome. The scores were obtained for the whole human proteome and used to encode mutation information in the known motif-containing protein regions. The scores range from 0 to 1 where 1 means that the given mutation is more likely to be pathogenic, while 0 that this substitution is more likely to be benign. As AlphaMissense prediction scores do not contain the scores for wildtype residue, we assigned a score equal to 0 for wildtype to wildtype substitution (as non-pathogenic). This results in 20 mutation pathogenicity scores, one for each amino acid mutation, for each position in a human protein. Using this data, we converted the mutation prediction scores into the PSSMs for human ELM instances from *ELM Dataset - All* by extracting the scores for the motif region of each protein. Next, the AlphaMissense scores were inverted (1-prediction score) to obtain higher scores for non-pathogenic substitutions at a given position in the PSSM. We obtained 1,989 AlphaMissense ELM instance PSSMs for 233 ELM classes. Finally, for each ELM class we generated a single AlphaMissense PSSM based on the multiple AlphaMissense ELM Instance PSSMs. We used aligned ELM instances so each of the ELM Instance PSSMs had identical size and the created ELM class PSSM had the same size. We calculated a single PSSM for ELM class by calculating the average of the mutation scores obtained for each amino acid at the given positions across all AlphaMissense ELM instance PSSMs belonging to the ELM class. Finally, the AlphaMissense PSSM for each ELM class was normalised to sum to 1 at each position (see *Methods - PSSM Normalisation*). The constructed AlphaMissense PSSMs for ELM class reflected the mutation profile derived from ELM instance information. We expected that the core motif positions in the PSSMs should have a lower tolerance for mutation, and tolerated substitutions at these positions would resemble the known motif preferences, and therefore the AlphaMissense PSSMs could reflect the positional amino acid preferences for a functional motif. We used CompariPSSM to benchmark if AlphaMissense PSSMs correlate with expected ELM classes (*ELM Instance PSSMs - All Dataset*) and encode the positional preferences correctly. We compared results obtained for AlphaMissense PSSMs to the results derived from the Conservation PSSMs benchmarking (see below).

## Conservation PSSMs Benchmarking

The conservation PSSM models for each ELM class in the *ELM Instance PSSMs - All Dataset* were derived from the protein alignments created using the Gopher algorithm [(4)](https://sciwheel.com/work/citation?ids=2637617&pre=&suf=&sa=0) using *Metazoa* proteomes (**Supplementary Table 1**). Firstly, all the motif instances were aligned for each motif class based on the ELM consensus with amino acid padding equal to 2. Next, for each aligned motif instance, the corresponding region in the protein alignment is translated to a frequency PSSM. If the alignment is not available for the motif-containing protein this instance is discarded. Finally, the conservation PSSM for each ELM class was obtained by calculating average scores across instance PSSMs and normalised to sum to 1 at each column (see *Methods - PSSM Normalisation*) in the same manner as described for the AlphaMissense PSSMs. This resulted in 238 ELM PSSMs. CompariPSSM was used to benchmark the conservation PSSMs against the *ELM Instance PSSMs - All Dataset* and the obtained results were compared with AlphaMissense PSSMs benchmarking

## Defined Positions for AlphaMissense and Conservation PSSMs

The AlphaMissense and Conservation PSSMs were investigated using motif positions that allow only one amino acid at a given position. These fixed positions were derived from ELM consensus by discarding the wildcard, prohibited and degenerate positions (i.e., allowing a group of amino acids) from the non-flexible length consensuses. Next, the defined positions were mapped to the AlphaMissense and Conservation PSSMs resulting in 448 fixed positions for 20 amino acids across 201 ELM classes and those columns were used to compare how both construction methods encode these residues. The fixed positions used in this comparison can be found in the Supplementary Table 7.

## ProP-PD Benchmarking

The *ProP-PD dataset* was benchmarked with a CompariPSSM analysis to compare the enriched motif binding determinants in the peptides returned for each selection against the set of motif class binding determinants in the *ELM Instance PSSMs - All Dataset*. Frequency PSSMs constructed from the 103 peptide alignments in the *ProP-PD dataset* were compared with the 243 frequency PSSMs constructed from the *ELM Instances PSSMs Dataset - All*. A predicted ELM class, computed as the highest-scoring match, was defined for each ProP-PD PSSM and compared to the expected ELM class for the selection from the *ProP-PD - Expected ELM Class* dataset (**Supplementary Table 2**). In the cases that there are more than one annotated ELM class for a bait, the highest ranking annotated ELM class according to the PSSM-PSSM comparison p-value for the bait is considered the correct prediction for that bait. The recall, precision, and Mann-Whitney p-value were calculated for ProP-PD - ELM class comparison results and the discriminatory ability was quantified using a ROC analysis.

## Motif Binding Determinant Clustering

The PSSM - PSSM comparison was applied to cluster Frequency PSSMs from the 90 ligand binding (LIG) class peptides set from the *ELM Instances PSSMs Dataset - All*. The -log_10_ PSSM - PSSM comparison p-value of each ELM class pair was calculated to create an all-by-all similarity matrix dataset. The matrix was then ordered based on hierarchical clustering using the Ward variance minimisation algorithm and the Euclidean distance as the distance metric between the classes, and plotted as a heatmap.

## Motif Annotation

A set of manually curated motif-containing peptides from human proteins were extracted from PDB [(8)](https://sciwheel.com/work/citation?ids=316245&pre=&suf=&sa=0). Peptides annotated in the ELM database were discarded and the remaining peptides were manually classified into an ELM class resulting in 208 peptides. An orthologue alignment was created for each peptide by running the GOPHER algorithm [(4)](https://sciwheel.com/work/citation?ids=2637617&pre=&suf=&sa=0) on the peptide-containing protein against a search database of metazoan proteomes (**Supplementary Table 1**). The aligned peptide-containing region was extracted from the alignment and was converted into a Frequency PSSM and compared with 269 frequency PSSMs constructed from the *ELM Instances Dataset - All* dataset with the PSSMSearch framework [(5)](https://sciwheel.com/work/citation?ids=5388579&pre=&suf=&sa=0).

[***Bibliography***](https://sciwheel.com/work/bibliography)

[1. Kumar M, Michael S, Alvarado-Valverde J, Mészáros B, Sámano-Sánchez H, Zeke A, et al. The Eukaryotic Linear Motif resource: 2022 release. Nucleic Acids Res. 2022 Jan 7;50(D1):D497–508.](https://sciwheel.com/work/bibliography/12175714)

[2. Benz C, Ali M, Krystkowiak I, Simonetti L, Sayadi A, Mihalic F, et al. Proteome-scale mapping of binding sites in the unstructured regions of the human proteome. Mol Syst Biol. 2022 Jan;18(1):e10584.](https://sciwheel.com/work/bibliography/12301587)

[3. Davey NE, Haslam NJ, Shields DC, Edwards RJ. SLiMFinder: a web server to find novel, significantly over-represented, short protein motifs. Nucleic Acids Res. 2010 Jul;38(Web Server issue):W534-9.](https://sciwheel.com/work/bibliography/5486474)

[4. Davey NE, Edwards RJ, Shields DC. The SLiMDisc server: short, linear motif discovery in proteins. Nucleic Acids Res. 2007 Jul;35(Web Server issue):W455-9.](https://sciwheel.com/work/bibliography/2637617)

[5. Krystkowiak I, Manguy J, Davey NE. PSSMSearch: a server for modeling, visualization, proteome-wide discovery and annotation of protein motif specificity determinants. Nucleic Acids Res. 2018 Jul 2;46(W1):W235–41.](https://sciwheel.com/work/bibliography/5388579)

[6. Altschul SF, Madden TL, Schäffer AA, Zhang J, Zhang Z, Miller W, et al. Gapped BLAST and PSI-BLAST: a new generation of protein database search programs. Nucleic Acids Res. 1997 Sep 1;25(17):3389–402.](https://sciwheel.com/work/bibliography/27750)

[7. Cheng J, Novati G, Pan J, Bycroft C, Žemgulytė A, Applebaum T, et al. Accurate proteome-wide missense variant effect prediction with AlphaMissense. Science. 2023 Sep 22;381(6664):eadg7492.](https://sciwheel.com/work/bibliography/15380065)

[8. Berman HM, Westbrook J, Feng Z, Gilliland G, Bhat TN, Weissig H, et al. The protein data bank. Nucleic Acids Res. 2000 Jan 1;28(1):235–42.](https://sciwheel.com/work/bibliography/316245)
